# Gas Chromatography/Mass Spectrometry-Based Metabolomic Profiling Reveals Alterations in Mouse Plasma and Liver in Response to Fava Beans

**DOI:** 10.1371/journal.pone.0151103

**Published:** 2016-03-16

**Authors:** Man Xiao, Guankui Du, Guobing Zhong, Dongjing Yan, Huazong Zeng, Wangwei Cai

**Affiliations:** 1 Department of Biochemistry and Molecular Biology, Hainan Medical College, Haikou, 571199, China; 2 Shanghai Sensichip Infotech Co., Ltd., Shanghai, 200433, China; Indian Institute of Chemical Technology, INDIA

## Abstract

Favism is a life-threatening hemolytic anemia resulting from the intake of fava beans by susceptible individuals with low erythrocytic glucose 6-phosphate dehydrogenase (G6PD) activity. However, little is known about the metabolomic changes in plasma and liver after the intake of fava beans in G6PD normal and deficient states. In this study, gas chromatography/mass spectrometry was used to analyze the plasma and liver metabolic alterations underlying the effects of fava beans in C3H- and G6PD-deficient (G6PDx) mice, and to find potential biomarkers and metabolic changes associated with favism. Our results showed that fava beans induced oxidative stress in both C3H and G6PDx mice. Significantly, metabolomic differences were observed in plasma and liver between the control and fava bean treated groups of both C3H and G6PDx mice. The levels of 7 and 21 metabolites in plasma showed significant differences between C3H-control (C3H-C)- and C3H fava beans-treated (C3H-FB) mice, and G6PDx-control (G6PDx-C)- and G6PDx fava beans-treated (G6PDx-FB) mice, respectively. Similarly, the levels of 7 and 25 metabolites in the liver showed significant differences between C3H and C3H-FB, and G6PDx and G6PDx-FB, respectively. The levels of oleic acid, linoleic acid, and creatinine were significantly increased in the plasma of both C3H-FB and G6PDx-FB mice. In the liver, more metabolic alterations were observed in G6PDx-FB mice than in C3H-FB mice, and were involved in a sugar, fatty acids, amino acids, cholesterol biosynthesis, the urea cycle, and the nucleotide metabolic pathway. These findings suggest that oleic acid, linoleic acid, and creatinine may be potential biomarkers of the response to fava beans in C3H and G6PDx mice and therefore that oleic acid and linoleic acid may be involved in oxidative stress induced by fava beans. This study demonstrates that G6PD activity in mice can affect their metabolic pathways in response to fava beans.

## Introduction

Glucose-6-phosphate dehydrogenase (G6PD, D-glucose-6-phosphate: NADP+ 1-oxidoreductase, E.C.1.1.1.49) is the enzyme that catalyzes the first reaction in the pentose phosphate pathway that converts glucose 6-phosphate (G6P) to 6-phosphogluconolactone, concomitantly producing the reduced form of nicotinamide dinucleotide hydrogen phosphate (NADPH) for reductive biosynthesis and oxidation reduction in the cell. NADPH enables cells to keep glutathione in the reduced form (GSH) and to detoxify hydrogen peroxide (H_2_O_2_) produced from oxygen radicals whenever cells are subjected to oxidative stress. Because the pentose phosphate pathway is the only source of NADPH in red blood cells (RBCs), G6PD activity is particularly crucial to prevent oxidative damage to hemoglobin and other essential molecules. Reactive oxygen species (ROS) play an important role in the pathophysiology of acute hemolytic anemia in G6PD-deficient patients[1–3]. G6PD deficiency leads to RBCs being highly vulnerable to oxidative damage owing to lack of NADPH, with limited capacity for regenerating GSH and displaying depletion of GSH. The enzyme deficiency can clinically cause neonatal jaundice, favism, drug- or infection-induced hemolytic crisis, and non-spherocytic hemolytic anemia[1–3].

Fava beans (*Vicia fava*), also called broad beans, are a valuable food resource because of their substantial and fairly well balanced protein content. Its high lysine content makes it a valuable nutritional supplement to cereals, although it contains less methionine[4]. The major drawback of fava beans comes from its hemotoxicity, which can give rise to favism in individuals with G6PD deficiency [5]. Fava beans contain high concentrations of the β-glycosides vicine and convicine, which can be converted by β-glycosidase to their respective aglycones divicine and isouramil. Hemotoxicity assays *in vitro* have demonstrated that divicine and isouramil are the components responsible for the oxidative damage to hemoglobin and the cell membrane [6–8]. It had been reported that divicine can induce favic response in G6PD-normal rats and produced hemolytic activity in rat RBCs [9–10]. Although the glycosides divicine and isouramil from fava beans have been proposed to be the components associated with hemolysis in favism, the metabolites of fava beans involved in triggering hemolysis *in vivo* are not entirely defined.

Metabolomics is the study of small biological molecules (i.e., metabolites) found within cells, tissues, and body fluids in response to environmental, pathogenic, and dietary changes or a genetic alteration. Endogenous metabolites are the most proximal reporters of alteration in the cells, tissue, and body in metabolic processes that respond to physiological or pathophysiological stimuli[11–12]. Therefore, measurement of metabolic changes in animal models in response to physiological or pathophysiological stimuli can be used to define the varying phenotypes observed. Many studies have employed high-throughput metabolite profiling to elucidate the biological pathways responsible for metabolic syndromes, nutrition, cancer, disease diagnosis, and drug metabolism[13–17].

To better understand the metabolic perturbations caused by fava beans possibly associated with favism, a comprehensive investigation in an animal model using high-throughput analysis such as metabolomics is necessary to identify the metabolites response to fava beans in liver and plasma samples. In this study, we employed a gas chromatography/mass spectrometry (GC/MS)-based metabolic profiling approach to compare the metabolites in plasma and liver tissue from G6PD-normal C3H mice and G6PD-deficient (G6PDx) mice, and to reveal alterations in metabolites as a result of the favic response.

## Materials and Methods

### Animal experiments

Animal experiments were carried out in strict accordance with the Guide for the Care and Use of Laboratory Animals of the National Institutes of Health. All the protocols and studies were approved by the Ethics Committee of Hainan Medical College for Animal Care and Use. For the care and use of animals utilized in this research, we monitored the animals twice per week and none of animals showed severe ill, died or moribund required for humane endpoints during the whole experiments. A protocol for early euthanasia/humane endpoints is performed if one of the following criteria is met: the loss of body weight more than 20%, a wound that cannot be improved after medication or animals developing neurological symptoms and unable to feed by themselves. For anesthesia and euthanasia/ humane endpoints, mice were treated with 2–3% of isoflurane and 3% of CO2 inhalation, respectively.

A total of 32 5- to 6-week-old male mice, including 16 C3H/HeJ (C3H) mice and 16 G6PD-deficient (G6PDx) mice on a C3H background[18], were obtained from the Model Animal Research Center of Nanjing University, China. All mice were housed in an environmentally controlled room at 22 ± 1°C with a relative humidity of 50 ± 5% under a light cycle of 12 h light/12 h dark. Food and tap water were provided *ad libitum*. Both C3H and G6PDx mice were randomly divided into a control group and a FB-treated group (n = 8 per group). The FB-treated mice underwent intragastric administration of fava bean flesh homogenate (20% (w/v)) at a dose of 10 g/kg body weight and the control mice received the same volume of water at intervals of 4 h for 32 h. At time points 0 (no fava beans or water ingestion), 1, 2, 4, 8, 12, 16, 20, 24, 28, and 32 h after the fava beans or water ingestion, blood samples were collected from the mouse tail for detecting the ROS, GSH, and malondialdehyde (MDA) content of RBCs. The mice were euthanized under chloral hydrate anesthesia when the RBC content of ROS plateaued. Plasma was separated by centrifugation at 3000xg at 4°C for 15 min and stored at −80°C for metabolomic analysis. Approximately 50-mg liver samples from the left lateral liver lobe were frozen for metabolomic analysis.

### Flow Cytometry

The level of intracellular ROS and GSH was determined by the alterations of fluorescence resulting from oxidation of dihydroethidium (DHE)[19] and Thiolite green (AAT Bioquest^®^, Inc., Sunnyvale, CA, USA). To determine the levels of ROS and GSH in the red blood cells, the RBCs from the mice with and without fava bean ingestion were diluted 10,000 times and incubated with 250 μM DHE and 50 μM Thiolite green at 37°C for 30 min. The excess fluorescent dyes were washed off with PBS. The levels of ROS and GSH in the RBCs were determined by a fluorescence-activated cell sorter (FACS Calibur, Becton Dickinson, Immunofluorometry systems, Mountain View, CA, USA). DHE or Thiolite green fluorescence was detected with excitation/emission at 518/605 nm (for DHE) or 490/520 nm (for Thiolite green). The mean fluorescence channel (MFC) of the entire RBC population was calculated for DHE and Thiolite Green by the FACS-equipped Cell Quest software.

### MDA assay

RBCs from citrated whole blood were washed three times with saline, and the MDA content of the red blood cell membrane measured using a MDA test kit (Nan jing Jian cheng Bioengineering Institute, Nangjing, China) according to the manufacturer’s instructions. The MDA content was calculated and presented as μmol/ g hemoglobin.

### Plasma sample pretreatment

Plasma samples were thawed at room temperature and vortex-mixed for 5 s. Then, 150 μL of methanol-chloroform solution (3:1, v/v) and 10 μL of L-2-chlorophenylalanine were added to 40 μL plasma. The mixture was vortexed for 30 s, then placed at −20°C for 20 min. The mixture was centrifuged at 14,000 g at 4°C for 15 min, then 160 μL of the supernatant was transferred to a GC vial and evaporated to dryness under a stream of nitrogen gas. Then, 30 μL of 20 mg/mL methoxyamine pyridine solution was added and reacted at 37°C for 90 min. Finally, 30 μL BSTFA reagent (containing 1% TMCS) was added into the mixture and reacted at 70°C for 60 min. After cooling to room temperature, 1 μL of the solution was injected into the GC/MS for analysis.

### Liver tissue sample pretreatment

The procedure for liver tissue sample pretreatment was performed as previously reported [20]. Briefly, for GC/MS analysis, 40 mg mouse liver tissue sample was submerged in 1.0 mL of water–methanol–chloroform solution (2:5:2, v/v/v), then 100 μL L-2-chlorophenylalanine added as an internal control. The mixture was then ultrasonicated in an ice bath for 60 min and then vortex mixed for another 2 min to extract metabolites and precipitate proteins. The precipitated proteins were separated by centrifugation at 14,000 g at 4°C for 15 min and 600 μL supernatant was collected separately from each sample into a vial (4 mL) with PTFE-lined screw cap. To concentrate and derivatize the metabolites in the solution, the supernatant was evaporated under nitrogen gas at 50°C to dryness. After the vial was dry, 30 μL of 20 mg/mL methoxyamine pyridine solution was added and reacted at 37°C for 90 min. Finally, 30 μL BSTFA reagent (containing 1% TMCS) was added into the mixture and reacted at 70°C for 60 min. After cooling to room temperature, 1 μL solution was injected into the GC-MS for analysis.

### Gas chromatography/mass spectrometry (GC/MS)

A HP 6890/5973 system coupled with a HP-5MS fused silica capillary column (30 m × 250 μm, 0.25 μm) was employed to profile metabolites. Helium (99.999%) was used as carrier gas with a flow rate of 1.0 mL per min, and 1 μL of sample was injected into the column with a splitless mode. The temperature of injection was set to 250°C. The column temperature was initially kept at 40°C for 3 min, then increased to 280°C at a rate of 8°C per min and maintained at 280°C for 3 min. The detector was a quadrupole mass spectrometer and the temperature of quadrupole and ion source were 150°C and 230°C, respectively. The data were acquired in full scan mode from m/z 45 to 800 and the acceleration voltage was turned on after a solvent delay of 3.0 min. Total ion chromatograms (TICs) and fragmentation patterns were acquired by GC/MSD ChemStation Software (Agilent, Shanghai, China). The software was used for metabolite identification by comparing the mass-to-charge ratios and the abundance of each compound detected against a standard mass chromatogram in the NIST (National Institute of Standards and Technology) mass spectral library. Peaks with a similarity index greater than 70% were tentatively identified as metabolites, whereas those having less than 70% similarity index were considered as unknown metabolites.

### Data processing and pattern recognition

All GC/MS raw files were processed by the R platform. The resulting tables were exported into Microsoft Excel, where normalization was performed prior to multivariate analyses. The resulting three-dimensional data matrix involving peak index (RT–m/z pair), sample names (observations), and normalized peak area percentage were submitted to the SIMCA-P software package (version 11.0, Umetrics, Umea, Sweden) for Principle component analysis (PCA), Partial Least Squares-Discriminant Analysis (PLS-DA), and Orthogonal Partial Least Squares-Discriminant Analysis (OPLS-DA). Unsupervised PCA was initially performed to explore dataset variations. PLS-DA and OPLS-DA were subsequently used to distinguish the differences between FB-treated groups and the control groups. The variable importance in the projection (VIP) values of all the metabolites from OPLS-DA model were taken as a criterion to find the variable importance of differential metabolites. Those variables with a VIP > 1.0 and difference with a *p*-value <0.05 were considered relevant for group discrimination. The statistical significance between two groups was evaluated by a univariate Student’s t-test. Statistical differences at *p* < 0.05 were considered significant. Two sample t-test statistics were used for the comparison of metabolite levels to determine the differences between the FB-treated and control groups.

## Results

### Fava beans induce oxidative stress in mice

**Fig 1:**
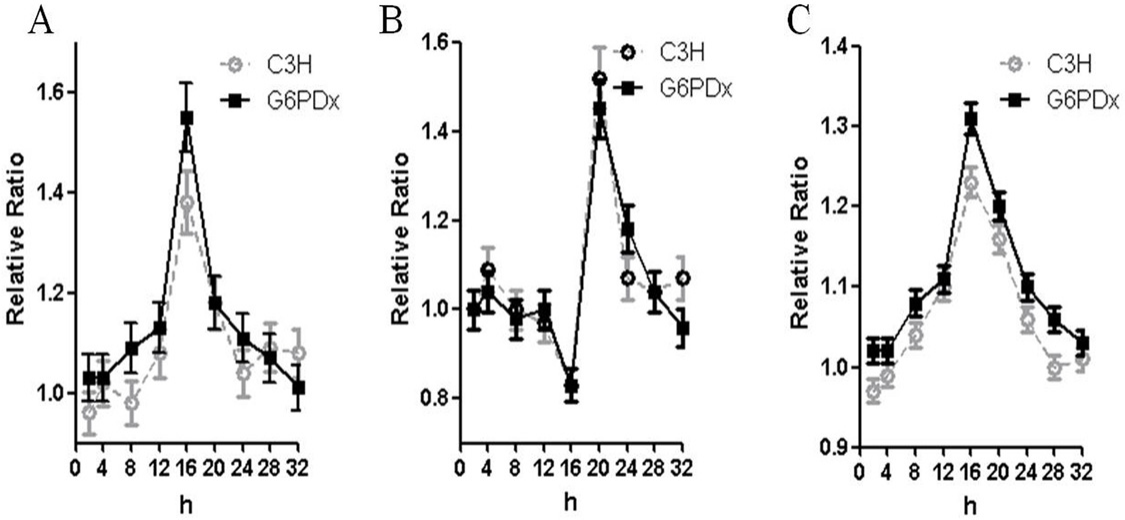
Kinetics of the changes of ROS, GSH, and MDA in response to fava beans in red blood cells of C3H and G6PDx mice. Results are presented as the relative ratios of ROS, GSH, and MDA in red blood cells of C3H-FB mice and G6PDx-FB mice to those in red blood cells of C3H control mice and G6PDx control mice, respectively. A. The relative ratio changes of ROS. B. The relative ratio changes of GSH. C. The relative ratio changes of MDA.

To determine whether the fava beans would induce biochemical alterations in mouse RBCs, we performed time-course analyses to evaluate the ROS, GSH, and MDA level of RBCs in G6PDx and C3H mice after intragastric administration of fava beans. Fig 1A clearly show that fava beans induced oxidative stress in both C3H and G6PDx mice (*p* < 0.05). The levels of ROS were significantly elevated, approximately 37% and 52% at 16 h in C3H-FB and G6PDx-FB mice, respectively, compared with the control and then decreased at 20 h. The production of ROS was also significantly higher at 16 h than that at the other time points in both C3H and G6PDx mice (*p* < 0.05). In contrast, the levels of GSH were significantly decreased at 16 h, then increased by about 46% and 43% at 20 h and then decreased at 24 h, respectively, in C3H and G6PDx mice (Fig 1B), correlating well with the ROS increase. Moreover, the levels of MDA were significantly elevated by about 23% and 31% at 16 h and then decreased at 20 h in C3H and G6PDx mice respectively (Fig 1C). The results suggest that ingestion of fava beans can trigger oxidative stress and lead to oxidative damage of lipids in mice, although a significant difference was not observed between C3H mice and G6PDx mice.

### Fava beans induce metabolic changes in the plasma

**Fig 2:**
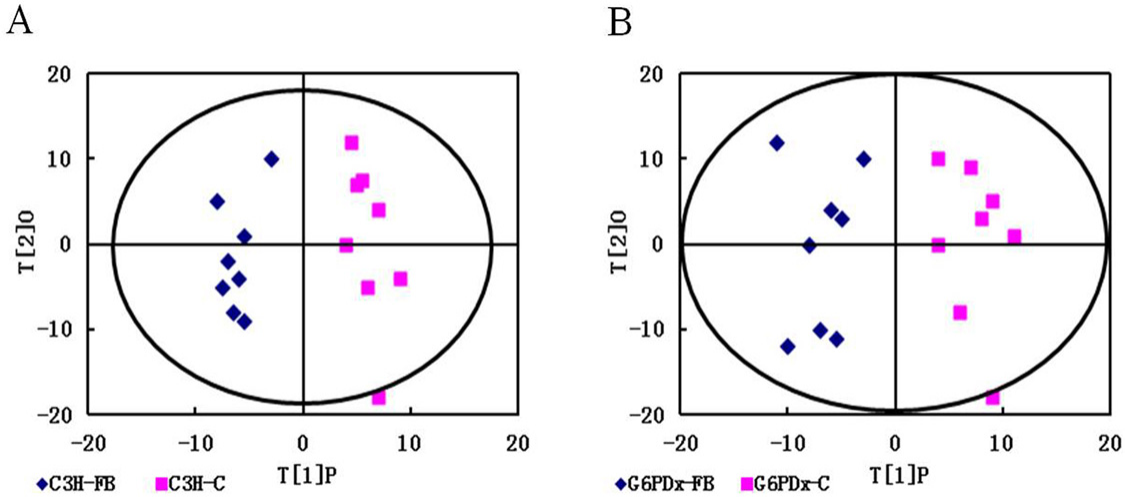
OPLS-DA scatter plots of GC/MS data of extracts from plasma of mice. Orthogonal partial least-squares discriminant analysis (OPLS-DA) score plots (t(1)P/ t(2)O) of the plasma of C3H group (A) and G6PDx group (B).

Representative GC/MS TIC chromatograms of plasma samples from the FB-treated and control groups in both G6PDx mice and C3H mice are shown in S1 Fig. After mean-centering, UV-scaling, and deconvolution, a total of 403 metabolites were detected in mouse plasma extracts by GC/MS. PCA results of the GC/MS data from the FB-treated and control groups are shown in S2 Fig. PCA indicated a clear distinction between the FB-treated and control groups in both C3H mice (R^2^X = 0.658) and G6PDx mice (R^2^X = 0.55). A PLS-DA scores plot showed the considerable separation achieved between FB-treated group and control group in both C3H mice (R^2^X = 0.854, Q^2^ = 0.328) and G6PDx mice (R^2^X = 0.913, Q^2^ = 0.68) (S3 Fig). Further analysis by the OPLS-DA model showed distinct discrimination between the metabolite profiles of the FB-treated and control groups in both C3H mice (R^2^X = 0.941, Q^2^ = 0.378) and G6PDx mice (R^2^X = 0.915, Q^2^ = 0.696) (Fig 2). The FB-treated group fell into the same region in both PLS-DA and OPLS-DA models with high R2Y values and Q2 values. These results indicate that there were significant alterations of the plasma metabolic profile among the FB-treated mice in comparison with the controls.

**Fig 3:**
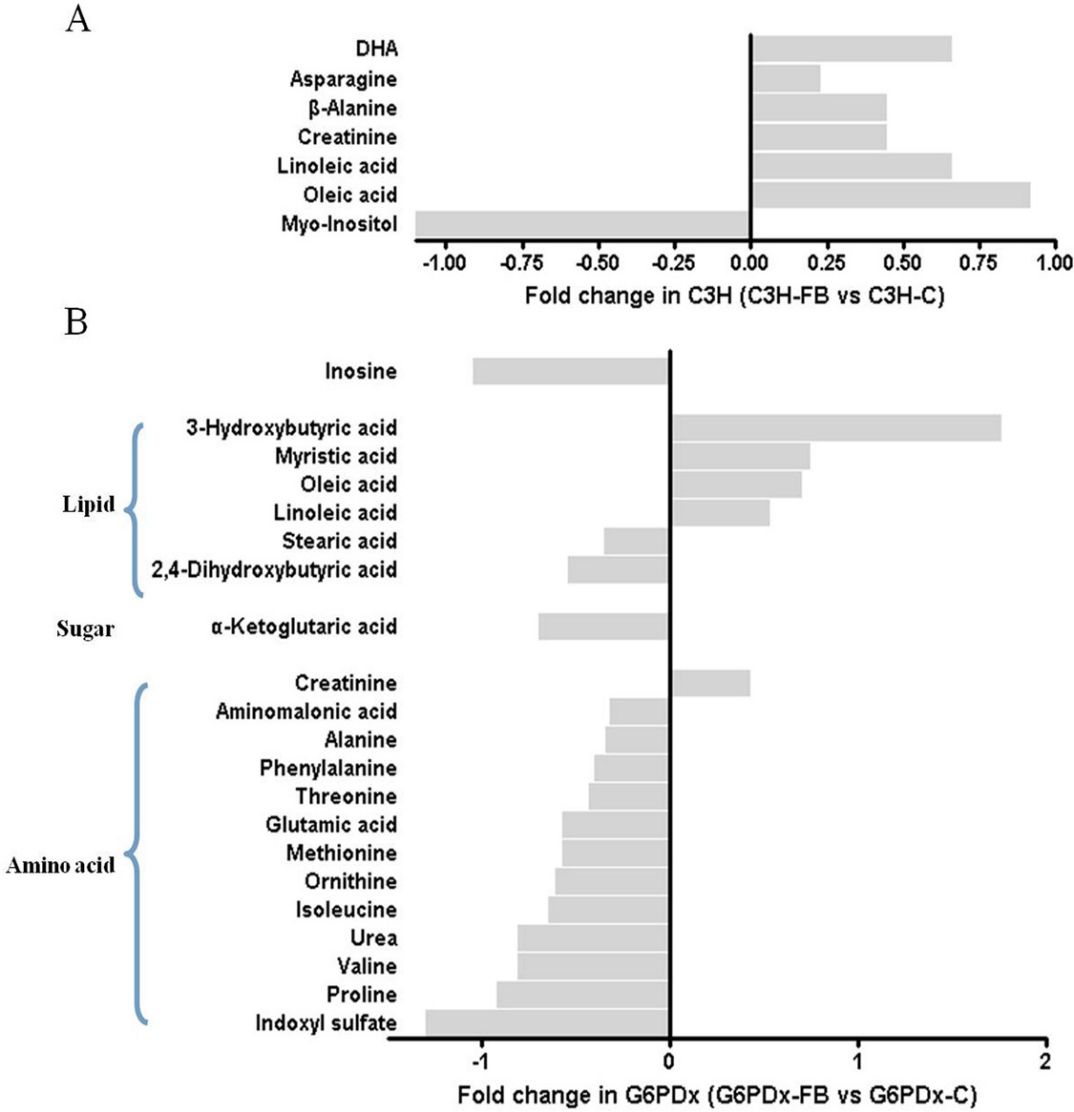
Relative levels of metabolites detected with significant changes in the plasma of the C3H-FB vs. C3H group and the G6PDx-FB vs. G6PDx group. Seven and 21 differential metabolites were significantly altered in C3H-FB mice compared with C3H control mice (A) and G6PDx-FB mice compared with G6PDx control mice (B) respectively.

The metabolic profiles of plasma showed that the differential metabolites in C3H-FB mice and G6PDx-FB mice were involved in the metabolism of carbohydrates, lipids, proteins, and purine (Tables 1 and 2 and Fig 3). Seven differential metabolites were found in C3H-FB mice compared with C3H control mice, including elevated β-alanine, asparagine, creatinine, DHA, oleic acid, linoleic acid, and decreased myo-inositol (Table 1; Fig 3A). However, 21 differential metabolites were significantly altered in G6PDx-FB mice compared with G6PDx control mice, including decreased indoxyl sulfate, inosine, α-ketoglutaric acid, alanine, ornithine, threonine, proline, phenylalanine, glutamic acid, isoleucine, valine, methionine, aminomalonic acid, 2,4-dihydroxybutyric acid, urea, and stearic acid, as well as increased creatinine, linoleic acid, oleic acid, myristic acid and 3-hydroxybutyric acid (Table 2 and Fig 3B). Among the differential metabolites, elevation of creatinine, oleic acid, and linoleic acid occurred in both C3H and G6PDx mice. Compared with C3H-FB mice, more striking alterations in amino acid metabolism were observed in the plasma of G6PDx-FB mice. The metabolites of protein, indoxyl sulfate, ornithine, urea, ketogenic and glucogenic amino acids (threonine, phenylalanine, and isoleucine), glucogenic amino acids (alanine, glutamic acid, proline, valine, and methionine) and aminomalonic acid, were significantly decreased in G6PDx mice (Table 2). The results showed clear different metabolic alterations in response to fava beans in plasma between G6PDx-FB mice and C3H-FB mice.

**Table 1:** The differential metabolites in the plasma between C3H-FB and C3H control mice.

No.	Compounds	NIST	VIP-Value	p-Value	Fold
		probability match(%)	(OPLS-DA)	(t-test)	Change
1	Myo-inositol	83.3	2.2	4.35E-03	−1.10
2	Oleic acid	87.5	1.98	1.28E-02	0.92
3	Linoleic acid	90.2	1.84	2.38E-02	0.66
4	Creatinine	89.6	1.76	3.28E-02	0.45
5	β-Alanine	79.5	1.81	2.63E-02	0.45
6	Asparagine	80.7	1.98	1.35E-02	0.23
7	DHA	88.3	1.97	1.40E-02	0.66

**Table 2:** The differential metabolites in the plasma between G6PDx-FB and G6PDx control mice.

No.	Compounds	NIST	VIP-Value	p-Value	Fold
		probability match(%)	(OPLS-DA)	(t-test)	Change
1	Indoxyl sulfate	82.3	2	9.98E-04	−1.30
2	Inosine	84.5	1.82	4.06E-03	−1.05
3	Proline^Δ^	81.7	2.1	3.36E-04	−0.92
4	Valine^Δ^	90.8	2.2	1.11E-04	−0.81
5	Urea	91.6	1.92	1.97E-03	−0.81
6	α-Ketoglutaric acid	87.4	1.42	3.60E-02	−0.70
7	Isoleucine*	91.5	2.02	8.19E-04	−0.65
8	Ornithine	86.1	1.77	5.64E-03	−0.61
9	Methionine^Δ^	93.2	2.06	5.37E-04	−0.57
10	Glutamic acid	89.5	1.64	1.23E-02	−0.57
11	2,4-Dihydroxybutyric acid	79.8	1.92	1.89E-03	−0.54
12	Threonine^Δ^	90.7	2.07	5.10E-04	−0.43
13	Phenylalanine*	88.6	2.1	3.52E-04	−0.40
14	Stearic acid	81.6	1.7	8.70E-03	−0.35
15	Alanine^Δ^	93.2	1.51	2.34E-02	−0.34
16	Aminomalonic acid	87.3	1.56	1.84E-02	−0.32
17	Creatinine	88.4	1.74	6.71E-03	0.43
18	Linoleic acid	89.7	1.47	2.83E-02	0.53
19	Oleic acid	92.4	1.6	1.45E-02	0.7
20	Myristic acid	87.3	1.5	2.48E-02	0.75
21	3-Hydroxybutyric acid	80.2	1.62	1.31E-02	1.76

Δ glucogenic amino acids

* glucogenic and ketogenic amino acids

### Fava beans induce metabolic changes in the liver

**Fig 4:**
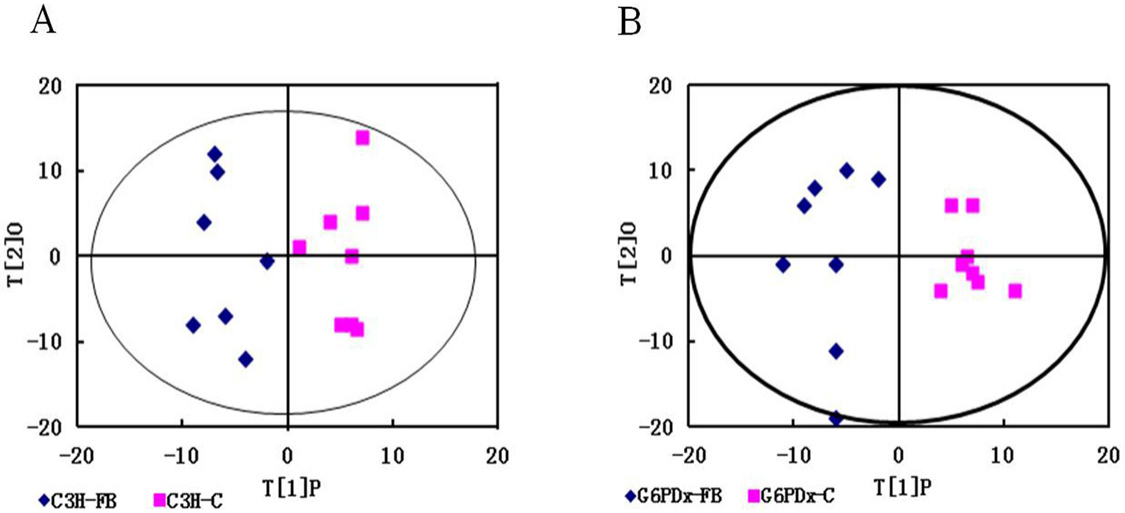
OPLS-DA scatter plots of GC/MS data of extracts from the liver of mice. Orthogonal partial least-squares discriminant analysis (OPLS-DA) score plots (t(1)P/ t(2)O) of the liver of C3H group (A) and G6PDx group (B).

Representative GC/MS TIC chromatograms of liver samples from the FB-treated and control groups in both C3H- and G6PD-deficient mice are shown in S4 Fig. After mean-centering, UV-scaling, and deconvolution, a total of 316 metabolites were detected in mouse liver extracts by GC-MS. PCA was first performed to identify the differences between the sample profiles. PCA indicated a clear distinction between the FB-treated group and control group in both C3H mice (R^2^X = 0.635) and G6PDx mice (R^2^X = 0.575). PLS-DA was carried out for class discrimination and biomarker identification. The PLS-DA scores plot showed the considerable separation achieved between the FB-treated group and control group in both C3H mice (R^2^X = 0.886, Q^2^ = 0.34) and G6PDx mice (R^2^X = 0.966, Q^2^ = 0.669). PCA and PLS-DA results are shown in S5 and S6 Figs. The OPLS-DA model was further performed to remove the structured noise and get more reliable metabolite information. The OPLS-DA models showed distinct discrimination between the metabolite profiles of the FB-treated and control groups in both C3H mice (R^2^X = 0.884, Q^2^ = 0.27) and G6PDx mice (R^2^X = 0.897, Q^2^ = 0.635) (Fig 4). The FB-treated group fell into the same region in both PLS-DA and OPLS-DA models with high R2Y values and Q2 values. These results indicate that there were significant variations in liver metabolites among the FB-treated mice compared with controls.

**Fig 5:**
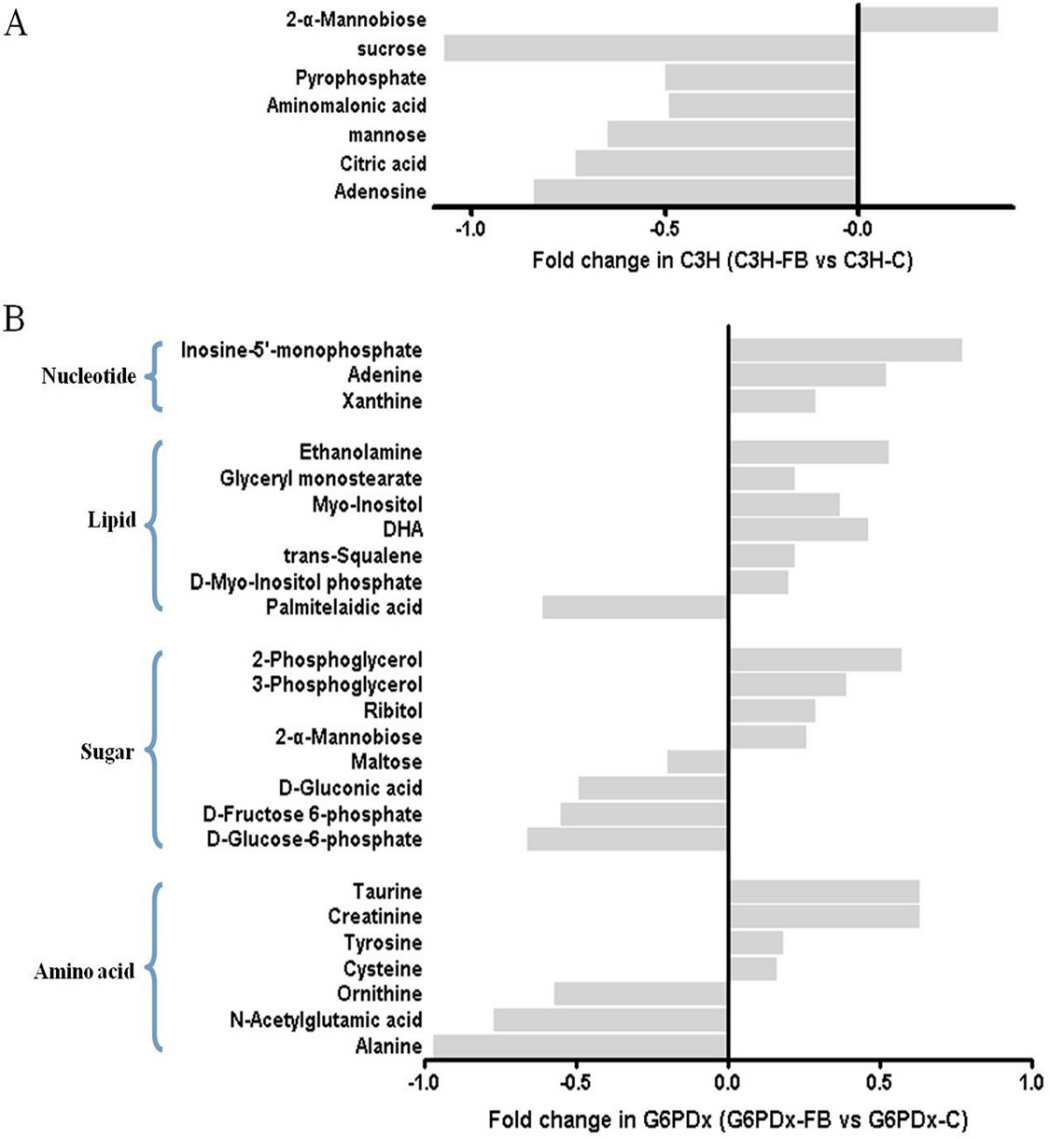
Relative levels of metabolites detected with significant changes in the liver of the C3H-FB vs. C3H group and the G6PDx-FB vs. G6PDx group. Seven and 25 differential metabolites were significantly altered in C3H-FB mice compared with C3H control mice (A) and G6PDx-FB mice compared with G6PDx control mice (B) respectively.

Table 3 and Fig 5A show the metabolic profiles of liver in FB-treated mice compared with the controls. The differential metabolites were involved in the metabolism of carbohydrate, lipids, nucleic acid, energy, and protein. Seven differential metabolites were found with significant differences in C3H-FB mice in comparison with the C3H controls, including decreased adenosine, citric acid, mannose, amino malonic acid, pyrophosphate, sucrose, and increased 2-α-mannobiose. In G6PDx-FB mice, 25 differential metabolites were detected with significant differences in comparison with the G6PDx controls, including increased D-myo-inositol phosphate, creatinine, cis-4,7,10,13,16,19-docosahexaenoic acid, glyceryl monostearate, ethanolamine, 2-phosphoglycerol, 3-phosphoglycerol, cysteine, tyrosine, taurine, trans-squalene, adenine, rititol, xanthine, inosine-5’-monophosphate, 2-α-mannobiose and decreased maltose, D-gluconic acid, D-fructose 6-phosphate, D-glucose-6-phosphate, alanine, ornithine, N-acetylglutamic acid, and palmitelaidic acid (Table 4 and Fig 5B). These findings suggest that more metabolic pathways were affected in response to fava beans in the liver of G6PDx-FB mice compared with C3H-FB mice.

**Table 3:** The differential metabolites in liver between C3H-FB and C3H control mice.

No.	Compounds	NIST	VIP-Value	p-Value	Fold
		probability match(%)	(OPLS-DA)	(t-test)	Change
1	Adenosine	91.5	1.83	2.91E-02	-0.84
2	Citric acid	88.5	1.95	1.83E-02	-0.73
3	Mannose	84.3	2.24	4.58E-03	-0.65
4	Aminomalonic acid	79.6	2.2	5.53E-03	-0.49
5	Pyrophosphate	80.1	2.18	6.43E-03	-0.5
6	Sucrose	90.5	1.89	2.33E-02	-1.07
7	2-α-Mannobiose	83.6	1.72	4.32E-02	0.36

**Table 4:** The differential metabolites in liver between G6PDx-FB and G6PDx control mice.

No.	Compounds	NIST	VIP-Value	p-Value	Fold
		probability match(%)	(OPLS-DA)	(t-test)	Change
1	Alanine^Δ^	89.5	1.75	2.54E-02	-0.97
2	D-Glucose-6-phosphate	92.7	1.34	7.49E-03	-0.66
3	Ornithine	90.1	1.73	3.87E-03	-0.57
4	D-Fructose 6-phosphate	91.3	1.25	1.42E-02	-0.55
5	D-Gluconic acid	85.6	1.84	4.21E-02	-0.49
6	N-Acetylglutamic acid	84.1	1.68	3.66E-03	-0.77
7	Palmitelaidic acid	79.6	1.32	2.20E-02	-0.61
8	Maltose	85.3	1.09	3.24E-02	-0.2
9	Cysteine^Δ^	90.8	1.25	3.90E-02	0.16
10	Tyrosine*	92.3	1.19	8.93E-03	0.18
11	D-Myo-Inositol phosphate	86.5	1.07	4.29E-03	0.2
12	Ribitol	90.7	1.12	4.19E-02	0.29
13	2-α-Mannobiose	83.7	1.26	2.33E-03	0.26
14	Inosine-5'-monophosphate	90.2	1.96	5.62E-06	0.77
15	Xanthine	85.4	1.8	4.99E-02	0.29
16	Adenine	87.3	1.65	2.51E-04	0.52
17	Myo-Inositol	91.8	2.02	5.72E-06	0.37
18	DHA	90.8	1.47	3.91E-02	0.46
	cis-4,7,10,13,16,19-Docosahexaenoic acid	79.5			
19	trans-Squalene	77.6	1.47	1.57E-03	0.33
20	Glyceryl monostearate	78.3	1.25	6.37E-03	0.22
21	2-Phosphoglycerol	85.6	1.48	6.42E-04	0.39
22	3-Phosphoglycerol	83.4	1.53	8.33E-04	0.57
23	Ethanolamine	70.8	1.46	1.93E-03	0.53
24	Creatinine	89.3	2.17	1.03E-05	0.63
25	Taurine	90.7	1.7	5.18E-03	0.63

Δ glucogenic amino acids

* glucogenic and ketogenic amino acids

## Discussion

We studied the kinetics of changes in the levels of ROS, GSH, and MDA in both C3H mice and G6PDx mice in response to fava beans. Our results showed that the contents of ROS and MDA were increased and that the depletion of GSH was well correlated with production of ROS during the favic response in both C3H and G6PDx mice. The results suggest that ingestion of fava beans can trigger oxidative stress and lead to oxidative damage of lipids in mice. However, we did not find any significant difference in the levels of ROS, GSH, and MDA between the C3H mice and G6PDx mice in response to fava beans. This may be owing to the G6PD activity in G6PDx mice not being low enough to produce an oxidative phenotype different from C3H mice, because the G6PD activity of erythrocytes in G6PDx mice is about 20% normal activity [18]. In human, it had been observed that only a few of G6PD deficient-individuals have hemolytic episode in response to fava beans though favism is associated with class Ⅱ(1%-10% of normal) and Ⅲ(10%-50% of normal) of G6PD deficiency, suggesting that the deficiency is a necessary but not sufficient cause of hemolysis [1–2,5].

One of the most important changes observed in this study is the increased levels of the unsaturated fatty acids, oleic acid, and linoleic acid, in the plasma of both G6PDx mice and C3H mice, suggesting that the lipid metabolic pathway was perturbed. Oleic and linoleic acids are the major components of lipoproteins and phospholipids in the cell membrane. The elevation of oleic acid and linoleic acid might indicate enhanced lipolysis or damage of the membrane, which may be associated with oxidative stress. It has been suggested that linoleic and oleic acids have antioxidant effects, protecting cells from oxidative stress[21]. However, the overall evidence of oleic acid and linoleic acid as antioxidants remains controversial. Oleic acid and linoleic acid might also exert pro-oxidant effects mediating peroxidation and ROS production. It had been shown that oleic acid and linoleic acid are more susceptible to lipid peroxidation and are considered to be the predominant substrate for lipid peroxidation processes [21–22]. Furthermore, oleic acid and linoleic acid may act as inducers of ROS production and enhance peroxidation and oxidative stress [23–30]. Linoleic acid can also decrease glutathione levels [31]. Our results show that the levels of oleic acid and linoleic acid were significantly elevated in plasma concomitantly with increased levels of ROS and MDA and depletion of GSH, which may be indicative of enhanced peroxidation and oxidative stress. These results indicate that oleic acid and linoleic acid may be potential metabolic biomarkers of the response to fava bean-induced oxidative stress in C3H and G6PDx mice.

In the liver, we observed significant changes of lipid metabolites in G6PDx-FB mice but not in C3H-FB mice, including *myo*-inositol, trans-squalene, fatty acids (glyceryl monostearate, DHA, and palmitelaidic acid), and ethanolamine. The changes in these lipids presumably reflect the dysregulation of lipid metabolism in the liver of G6PDx-FB mice. These findings indicate that the metabolism of lipids in the liver is different between C3H mice and G6PDx mice in response to fava beans.

**Fig 6:**
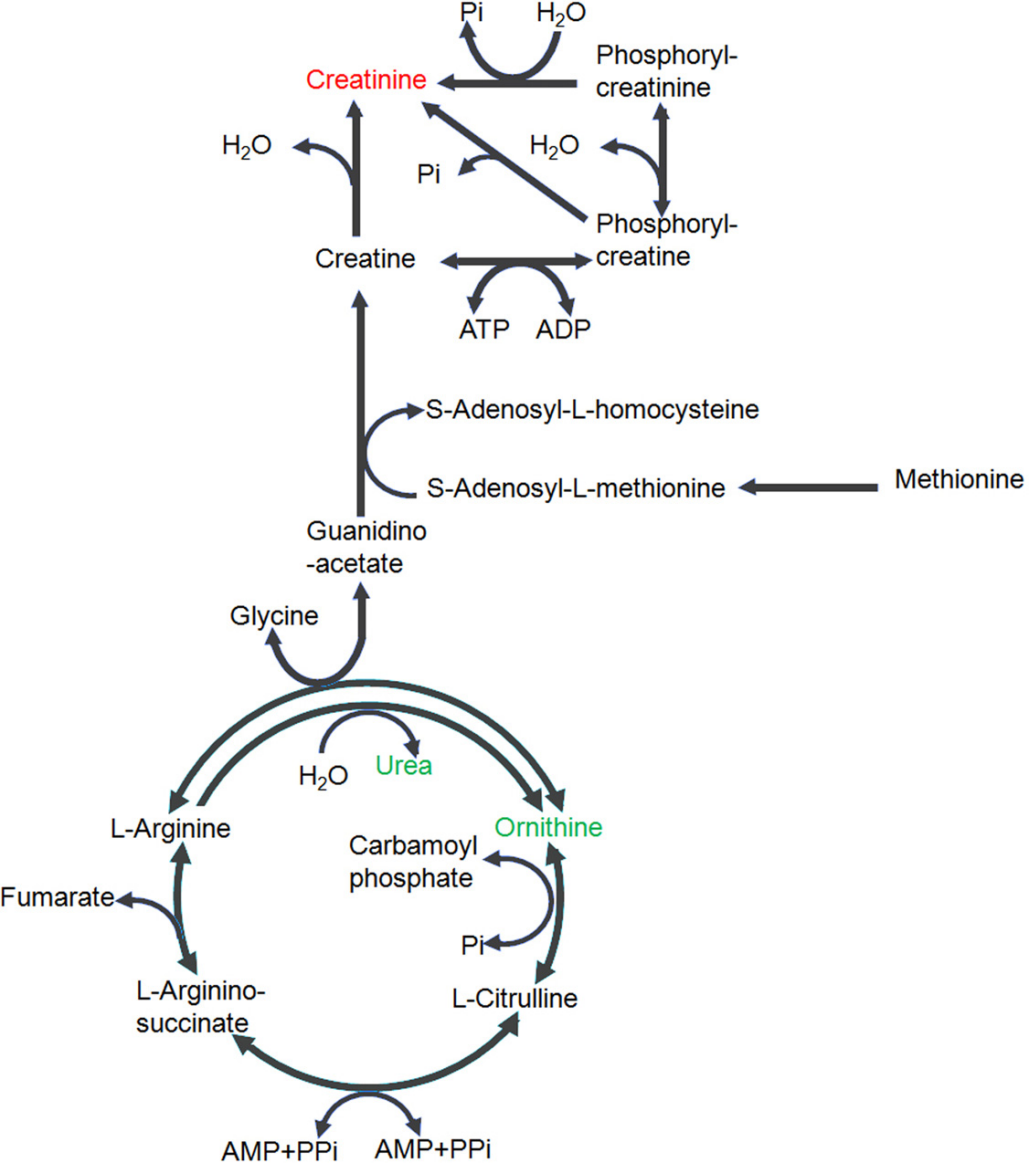
Effects of fava beans on metabolic pathways of the urea cycle and creatine according to the KEGG PATHWAY database. Putative metabolic pathways were inferred from changes in the plasma and liver levels of intermediates in the G6PDx-FB mice. Red indicates the significantly elevated metabolites relative to those in the controls. Green indicates significantly reduced metabolites relative to those of the controls.

It can also be noted that significant alterations in creatine metabolism and the urea cycle were observed in the plasma of G6PDx mice in comparison with C3H mice. In G6PDx mice, we observed a significant increase in creatinine and decrease in ornithine and urea. Creatinine, ornithine, and urea are the products of L-arginine via different metabolic shunts[32] (Fig 6). Creatinine is the non-enzymatic degradation product of creatine and phosphocreatine, which play a fundamental role in the transfer of energy from mitochondria to cytosol, while ornithine and urea are the metabolites involved in urea cycle. The changes in creatinine, ornithine, and urea indicated that creatinine synthesis from L-arginine via creatine was up-regulated or more phosphocreatine was hydrolyzed to release energy, and the urea cycle was down-regulated in G6PDx mice in response to fava beans. Of interest, we also observed that creatinine was increased and ornithine was significantly decreased in the liver of G6PDx-FB mice although urea was not changed, consistent with the changes in plasma, confirming that the metabolism of creatine or phosphocreatine and urea cycle were affected in G6PDx-FB mice.

**Fig 7:**
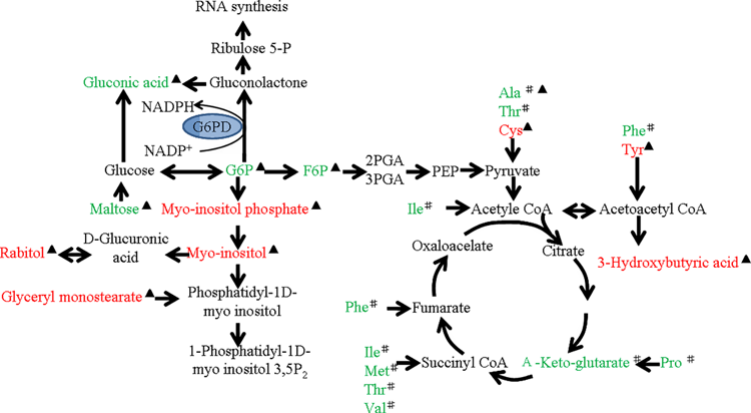
Effects of fava beans on metabolic pathways according to the KEGG PATHWAY database. Putative metabolic pathways were inferred from changes of the intermediates levels in the liver and plasma of the G6PDx-FB mice. Red indicates the significantly elevated metabolites relative to those in the controls. Green shows significantly reduced metabolites relative to those in the controls.

It had been reported that pentose phosphate pathway flux increased in human G6PD normal RBCs and the glycolysis pathway was enhanced in G6PD-deficient RBCs under diamide-induced oxidative stress [33]. The glycolysis pathway is also enhanced in G6PD-deficient hepatoma cells under diamide-induced oxidative stress[34]. We found that some differential metabolites of energy metabolism in the liver were different between C3H mice and G6PDx mice (Table 3 and Fig 5A). Decreased levels of citric acid and pyrophosphate were observed in C3H-FB mice, whereas decreased levels of D-glucose-6-phosphate and D-fructose-6-phosphate and elevated levels of 2-phosphoglycerol and 3-phosphoglycerol were found in G6PDx-FB mice, indicating that the affected pathways in C3H-FB mice are different from those in the G6PDx-FB mice in response to fava beans. Citric acid, adenosine, and pyrophosphate are involved respectively in tricarboxylic acid cycle (TCA cycle) and ATP hydrolysis. The significant declines of citric acid, adenosine, and pyrophosphate in C3H mice suggest that the TCA cycle and ATP hydrolysis are down-regulated and that probably more glucose enters the pentose phosphate pathway under fava bean-induced oxidative stress, although we did not find the changed metabolites such as ribose-5-phosphate and sedoheptulose-7-phosphate in the pentose phosphate pathway as described in human red blood cells[33]. In G6PDx-FB mice, the changes of glycolytic pathway metabolites, decreased levels in glucose-6-phosphate and fructose-6-phosphate suggest that the entry of glucose into glycolysis may be enhanced (Fig 7). These changes of metabolic pathways in energy metabolism could be related to the G6PD deficiency, which results in reduced flux of glucose into pentose phosphate pathway. The findings are to a some extent in agreement with the recent findings on the metabolic changes associated with enhanced glucolysis in G6PD-deficient hepatoma cells [34] and human G6PD-deficient RBCs in response to diamide [33]. In addition, the levels of inosine-5'-monophosphate (IMP), adenine, and xanthine were increased, which corresponds well with the increased energy metabolism induced by oxidative stress found in human erythrocytes [33, 35], suggesting that excessive ATP use occurred in the liver in response to fava bean-induced oxidative stress. Our findings indicate that metabolic pathways in energy metabolism in the liver were differentially perturbed in response to fava beans in C3H mice and G6PDx mice.

**Fig 8:**
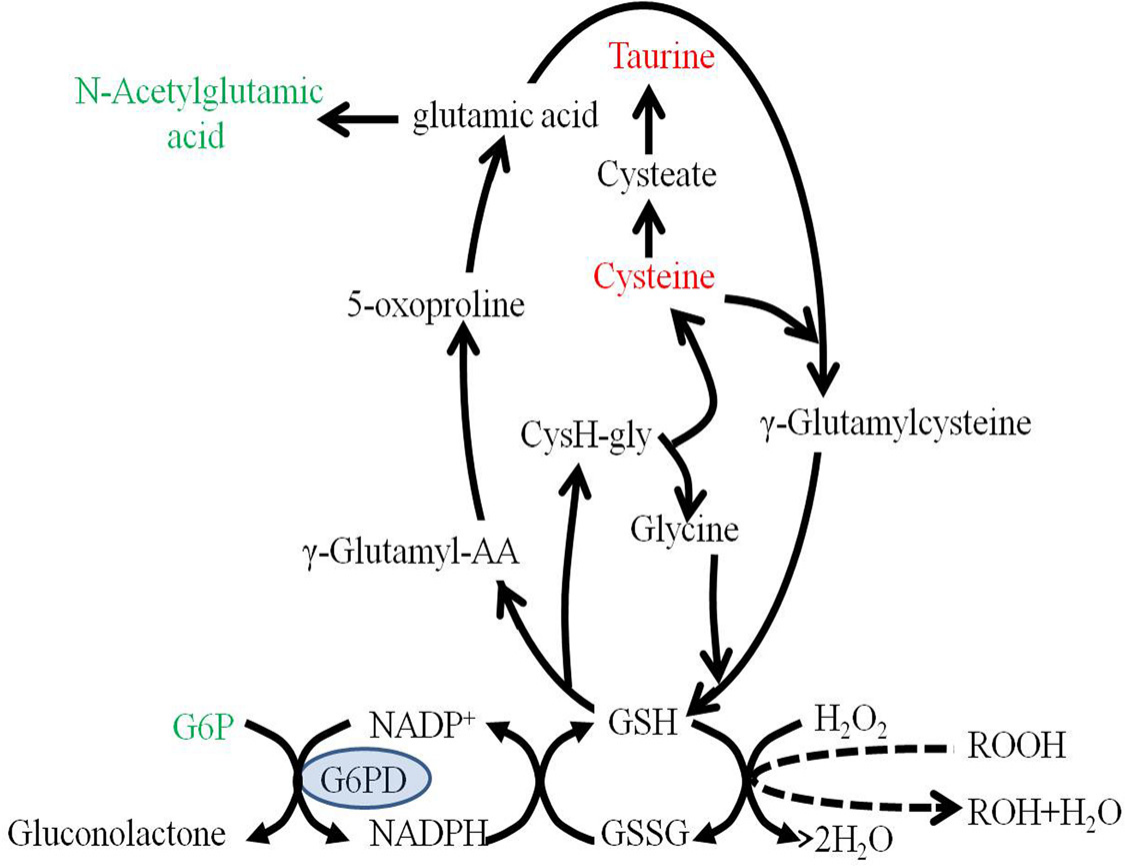
The alteration of cysteine and its relevant metabolic pathways according to the KEGG PATHWAY database. Putative metabolic pathways were inferred from changes in the intermediates levels in the liver of the G6PDx-FB mice. Red indicates the significantly elevated metabolites relative to those in the controls. Green shows significantly reduced metabolites relative to those in the controls.

Differential significant changes in the metabolites of amino acids were found in plasma and liver in both C3H-FB mice and G6PDx-FB mice in response to fava beans. In plasma, more striking alterations in amino acid metabolism were observed in G6PDx mice compared with C3H mice. We observed a significant decrease in glucogenic amino acids and glucogenic and ketogenic amino acids along with an increase in 3-hydroxybutyratethe, one compound of the ketone bodies, suggesting that these amino acids may be transformed to ketone bodies via the ketone bodies pathway or to glucose via the gluconeogenesis pathway (Fig 7). These results implied that the cellular pathways associated with some glucogenic amino acids, and glucogenic and ketogenic amino acids could be perturbed and the differential amino acids may be potential biomarkers in plasma in response to fava beans in G6PDx mice. In the liver, the differential amino acids involved in cysteine metabolism were found in both C3H-FB mice and G6PDx-FB mice, which were different from those in the plasma of both C3H mice and G6PDx mice. Cysteine and taurine were increased in the liver of G6PDx-FB mice, whereas aminomalonic acid was decreased in the liver of C3H-FB mice. Cysteine can come from the degradation of protein and GSH or from methionine metabolism[36], and taurine is the product of cysteine metabolism[37–38]. It had been reported that the elevated level of cysteine is related to depletion of glutathione metabolism and oxidative stress induced by diamide in G6PD-deficient hepatoma cells [34].The elevation of cysteine and taurine may reflect the dysregulation of GSH metabolism in G6PDx-FB mice in response to fava bean-induced oxidative stress (Fig 8). Aminomalonic acid is a dicarboxylic acid that could be derived via β-elimination of sulfur from cysteine and the product of oxidative damage to amino acid residues in proteins[39]. The decrease of aminomalonic acid indicates that the flux of cysteine into the GSH synthesis pathway may be enhanced in the liver of C3H mice with normal G6PD activity compared with G6PDx mice.

In this study, we found that the changes in many metabolites in the liver were inconsistent with their levels in plasma in both C3H and G6PDx mice. This may be due to the plasma metabolic profile reflecting systemic metabolic effects in response to fava beans from all cells in the mice, while the liver metabolic profile only demonstrates metabolism related to the response to fava beans in the liver.

In summary, our results demonstrate that fava beans induced oxidative stress in both C3H and G6PDx mice. Significant metabolomic differences in plasma and liver were observed among the C3H-FB mice, G6PDx-FB mice, and their controls. The results also indicate that some significantly changed metabolites may be potential biomarkers of the response to fava beans, and moreover, may be associated with oxidative stress. Further studies on the plasma metabolic profile of favic patients might better elucidate the precise metabolic changes occurring in favism.

## Supporting Information

S1 Fig The representative GC/MS chromatograms of plasma samples from No.3 of C3H-C group, No.4 of C3H-FB group, No.2 of G6PDx-C group and No. 5 of G6PDx-FB group. X-axis is retention time (min); Y-axis is intensity of MS (%).
(TIF)

S2 Fig PCA scatter plots of GC/MS data of extracts from plasma of mice. Principal Component Analysis (PCA) score plots (t(1)P/ t(2)O) of the plasma of C3H (A) group and G6PDx group (B).
(TIF)

S3 Fig PLS-DA scatter plots of GC/MS data of extracts from plasma of mice. Orthogonal partial least-squares discriminant analysis (OPLS-DA) score plots (t(1)P/ t(2)O) of the **plasma** of C3H (A) group and G6PDx group (B).
(TIF)

S4 Fig The representative GC/MS chromatograms of tissue liver samples from No.3 of C3H-C group, No.4 of C3H-FB group, No.4 of G6PDx-C group and No.4 of G6PDx -FB group. X-axis is retention time (min); Y-axis is intensity of MS (%).
(TIF)

S5 Fig PCA scatter plots of GC/MS data of extracts from tissues Liver of mice. Principal Component Analysis (PCA) score plots (t(1)P/ t(2)O) of the liver of C3H (A) group and G6PDx group (B).
(TIF)

S6 Fig PLS-DA scatter plots of GC/MS data of extracts from tissues Liver of mice. Orthogonal partial least-squares discriminant analysis (OPLS-DA) score plots (t(1)P/ t(2)O) of the liver of C3H (A) group and G6PDx group (B).
(TIF)

S7 Fig The *R*^*2*^ *and Q*^*2*^ results of random permutation over at least n = 500 of GC/MS data of extracts from plasma of mice. (A) C3H group and (B) G6PDx group.
(TIF)

S8 Fig The e *R*^*2*^ *and Q*^*2*^ results of random permutation over at least n = 500 of GC/MS data of extracts from liver of mice. (A) C3H group and (B) G6PDx group.
(TIF)

## Abbreviations

G6PD: glucose 6-phosphate dehydrogenase
G6PDx: G6PD-deficient
NADPH: nicotinamide dinucleotide hydrogen phosphate
GSH: reduced glutathione
H2O2: hydrogen peroxide
RBCs: red blood cells
ROS: Reactive oxygen species
GC/MS: gas chromatography/mass spectrometry
MDA: malondialdehyde
DHE: dihydroethidium
