# Supplementary figures and images for: Gas Chromatography/Mass Spectrometry-Based Metabolomic Profiling Reveals Alterations in Mouse Plasma and Liver in Response to Fava Beans
Source: PLoS One. 2016 Mar 16;11(3):e0151103. doi: 10.1371/journal.pone.0151103 (PMC4794218; doi:10.1371/journal.pone.0151103)

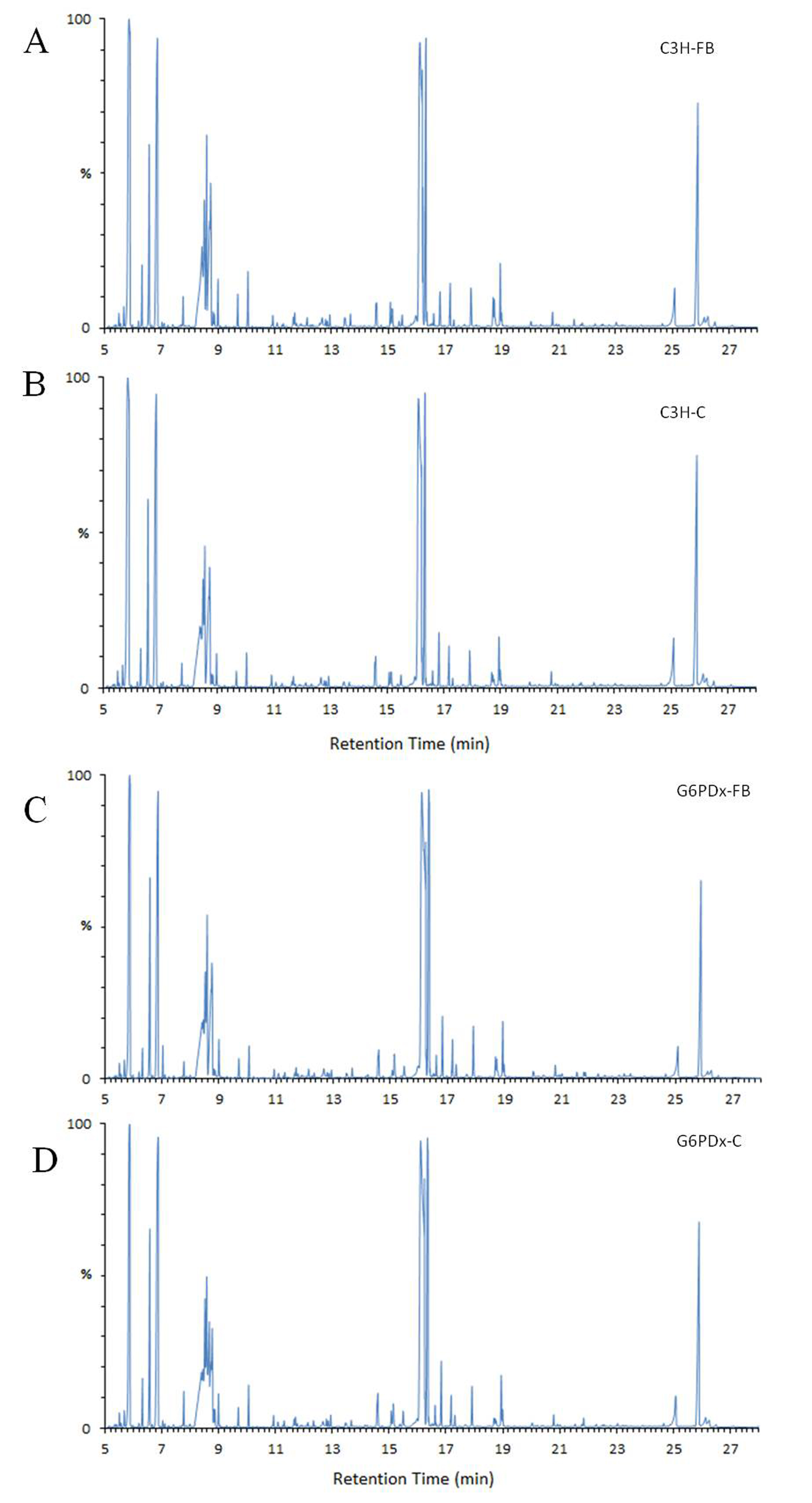

Supplement: S1 Fig — X-axis is retention time (min); Y-axis is intensity of MS (%). (TIF) [file pone.0151103.s001.tif]

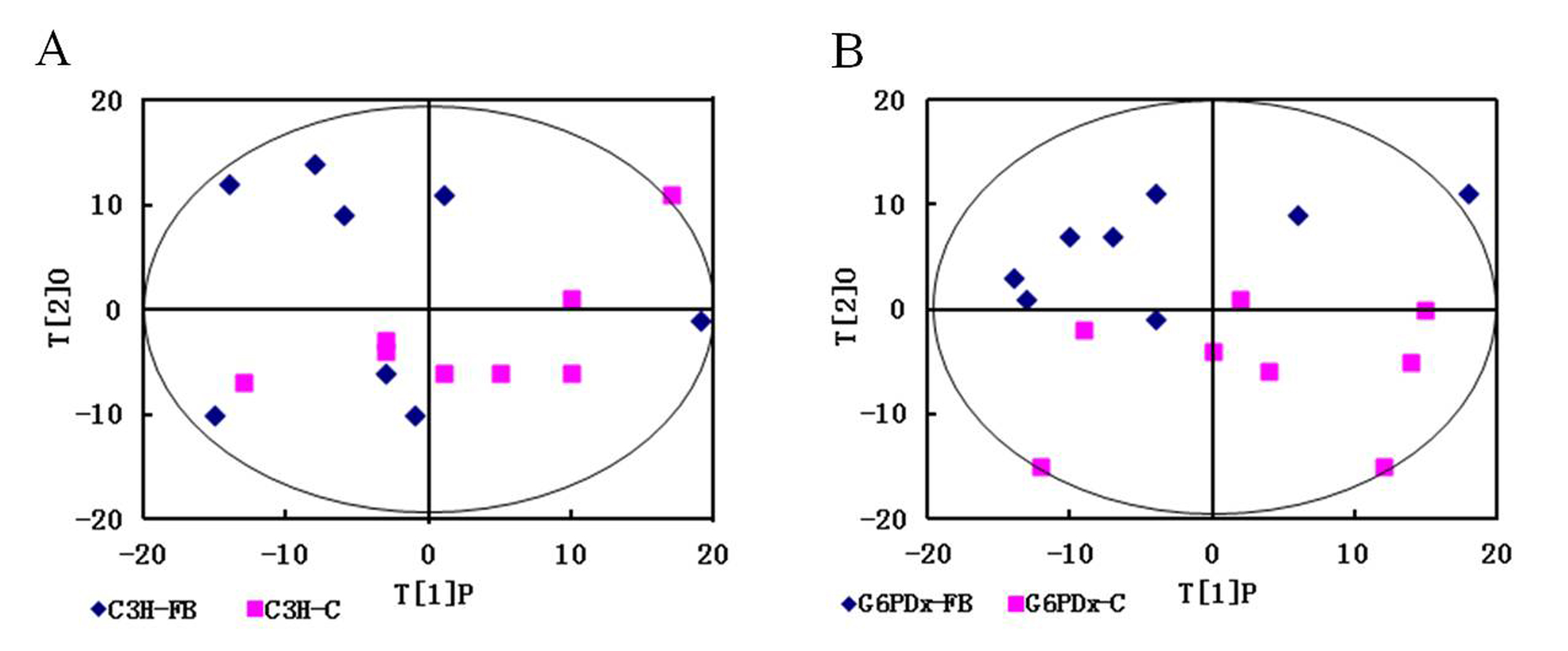

Supplement: S2 Fig — Principal Component Analysis (PCA) score plots (t(1)P/ t(2)O) of the plasma of C3H (A) group and G6PDx group (B). (TIF) [file pone.0151103.s002.tif]

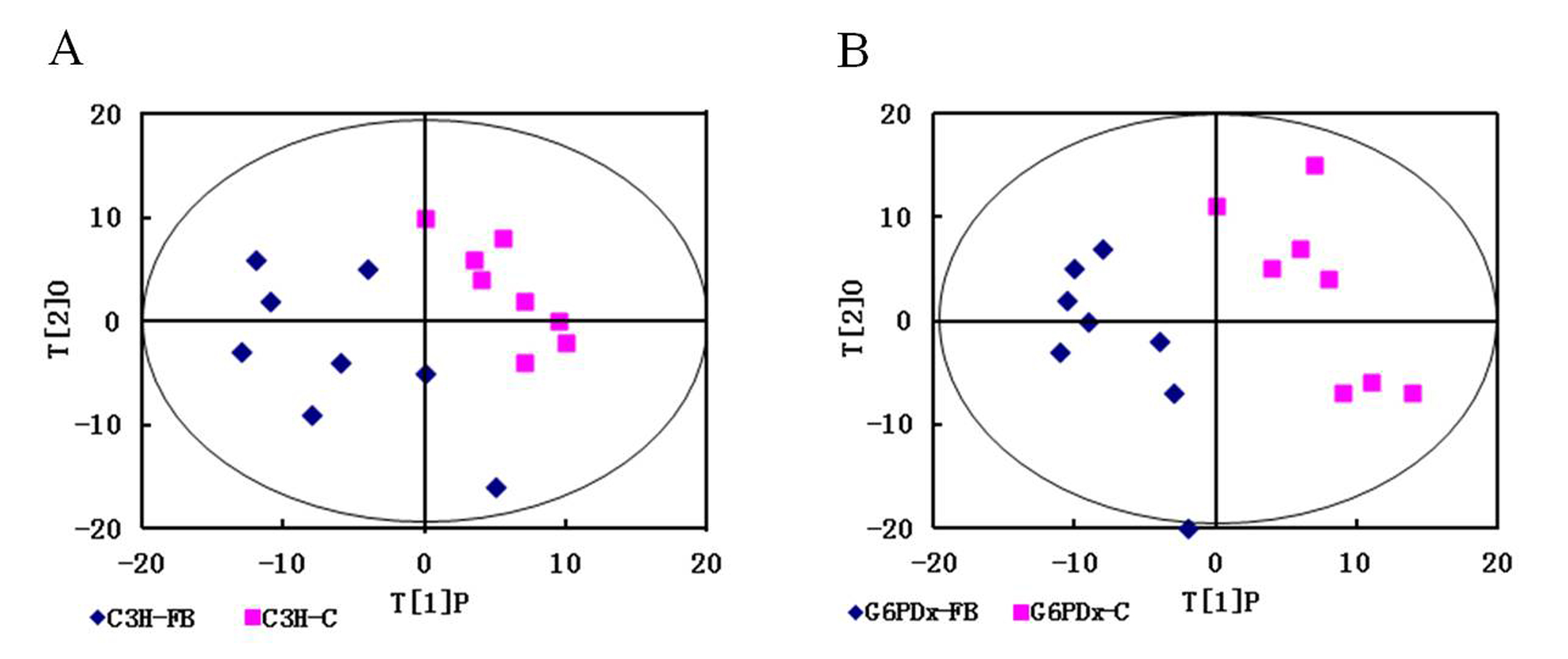

Supplement: S3 Fig — Orthogonal partial least-squares discriminant analysis (OPLS-DA) score plots (t(1)P/ t(2)O) of the plasma of C3H (A) group and G6PDx group (B). (TIF) [file pone.0151103.s003.tif]

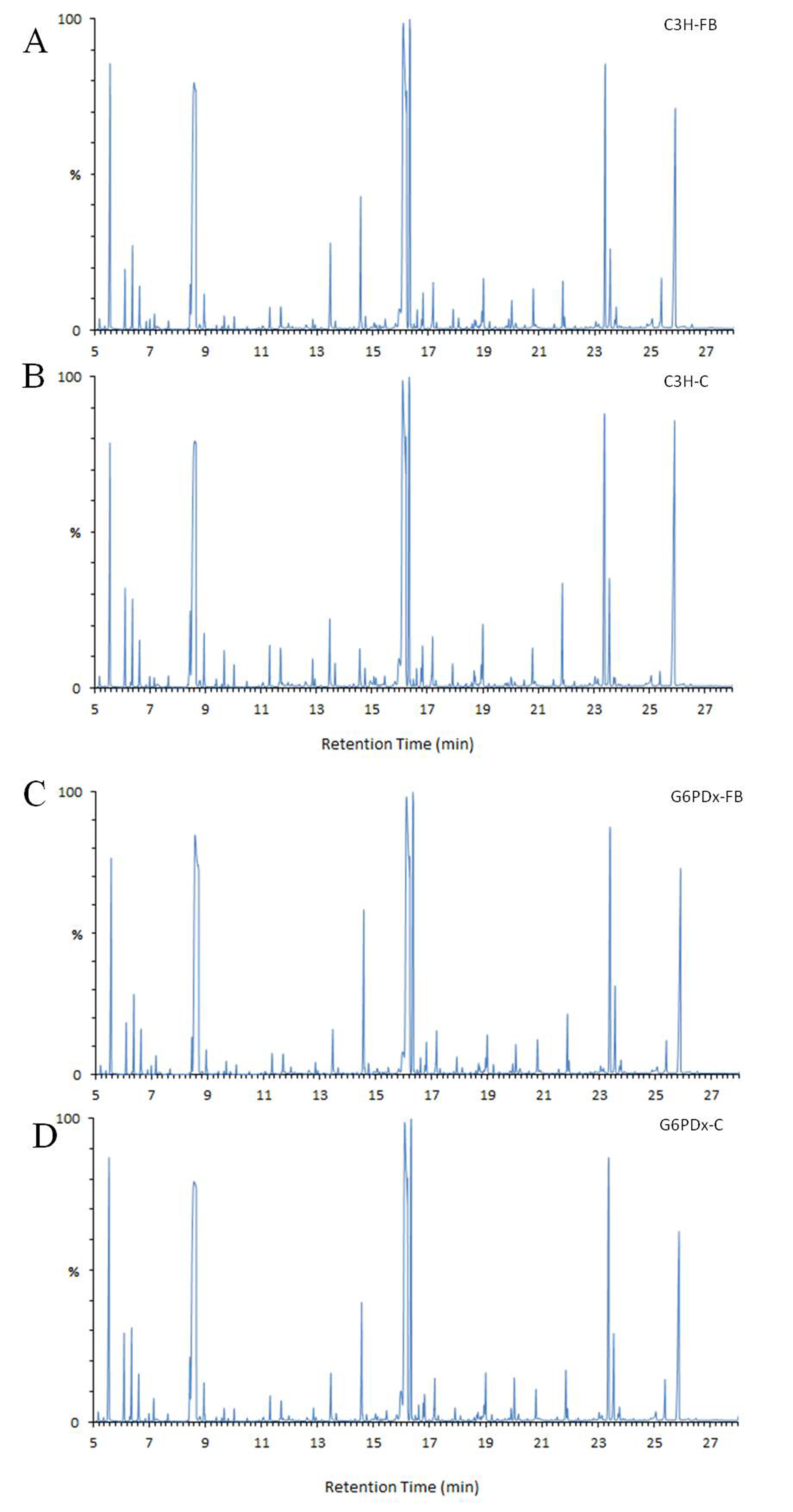

Supplement: S4 Fig — X-axis is retention time (min); Y-axis is intensity of MS (%). (TIF) [file pone.0151103.s004.tif]

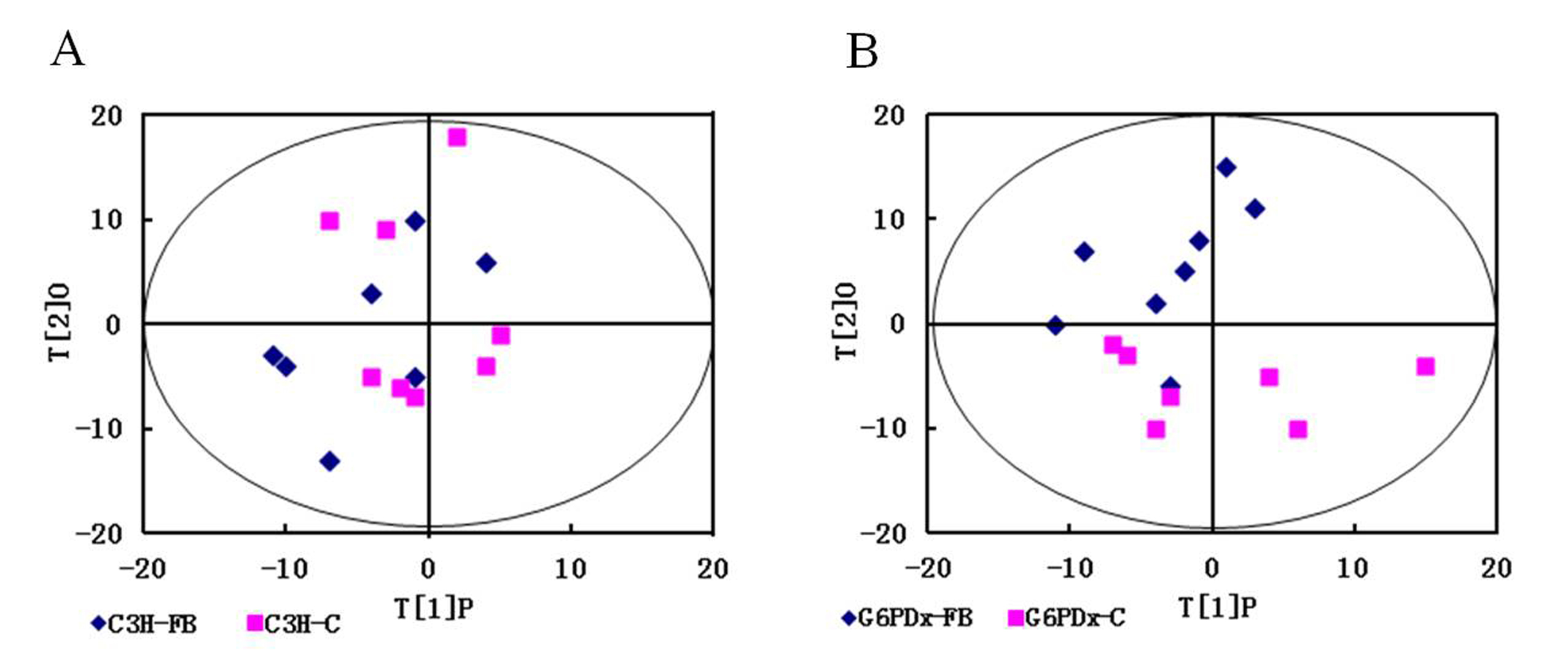

Supplement: S5 Fig — Principal Component Analysis (PCA) score plots (t(1)P/ t(2)O) of the liver of C3H (A) group and G6PDx group (B). (TIF) [file pone.0151103.s005.tif]

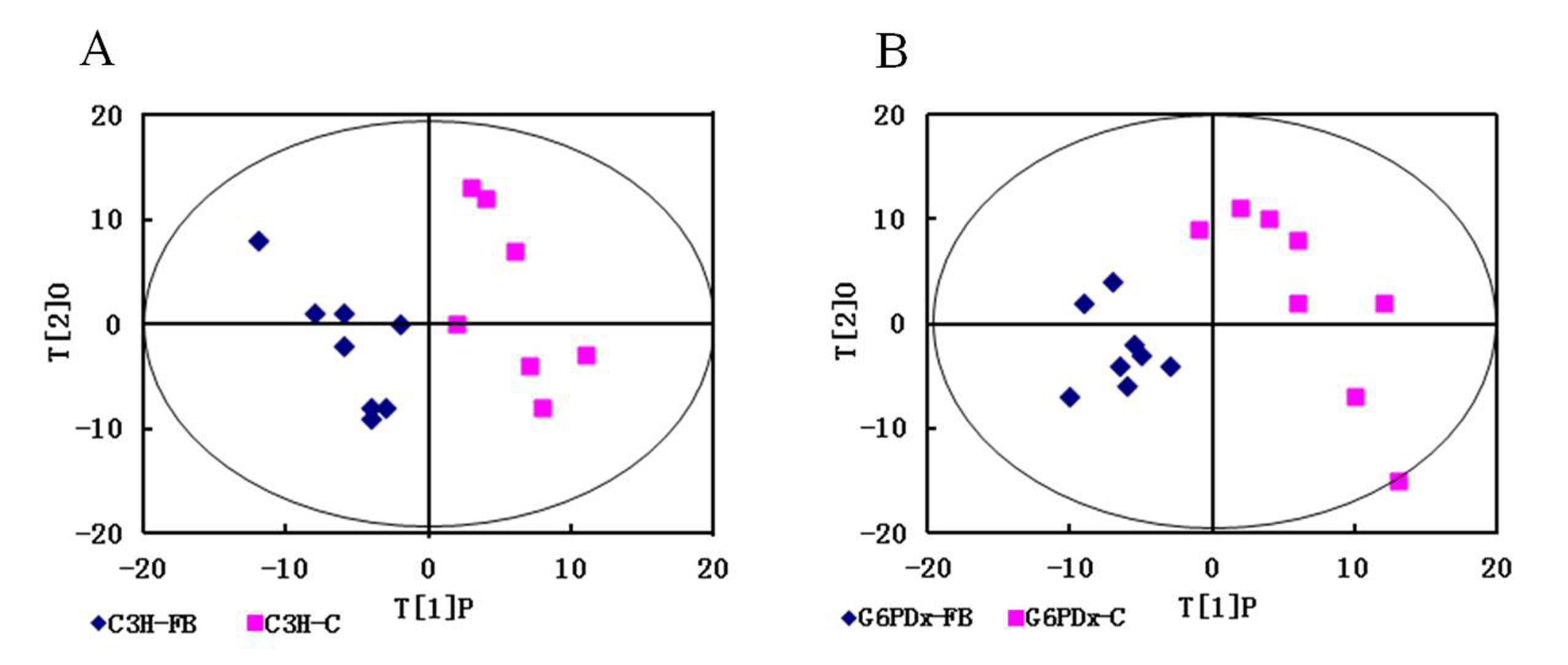

Supplement: S6 Fig — Orthogonal partial least-squares discriminant analysis (OPLS-DA) score plots (t(1)P/ t(2)O) of the liver of C3H (A) group and G6PDx group (B). (TIF) [file pone.0151103.s006.tif]

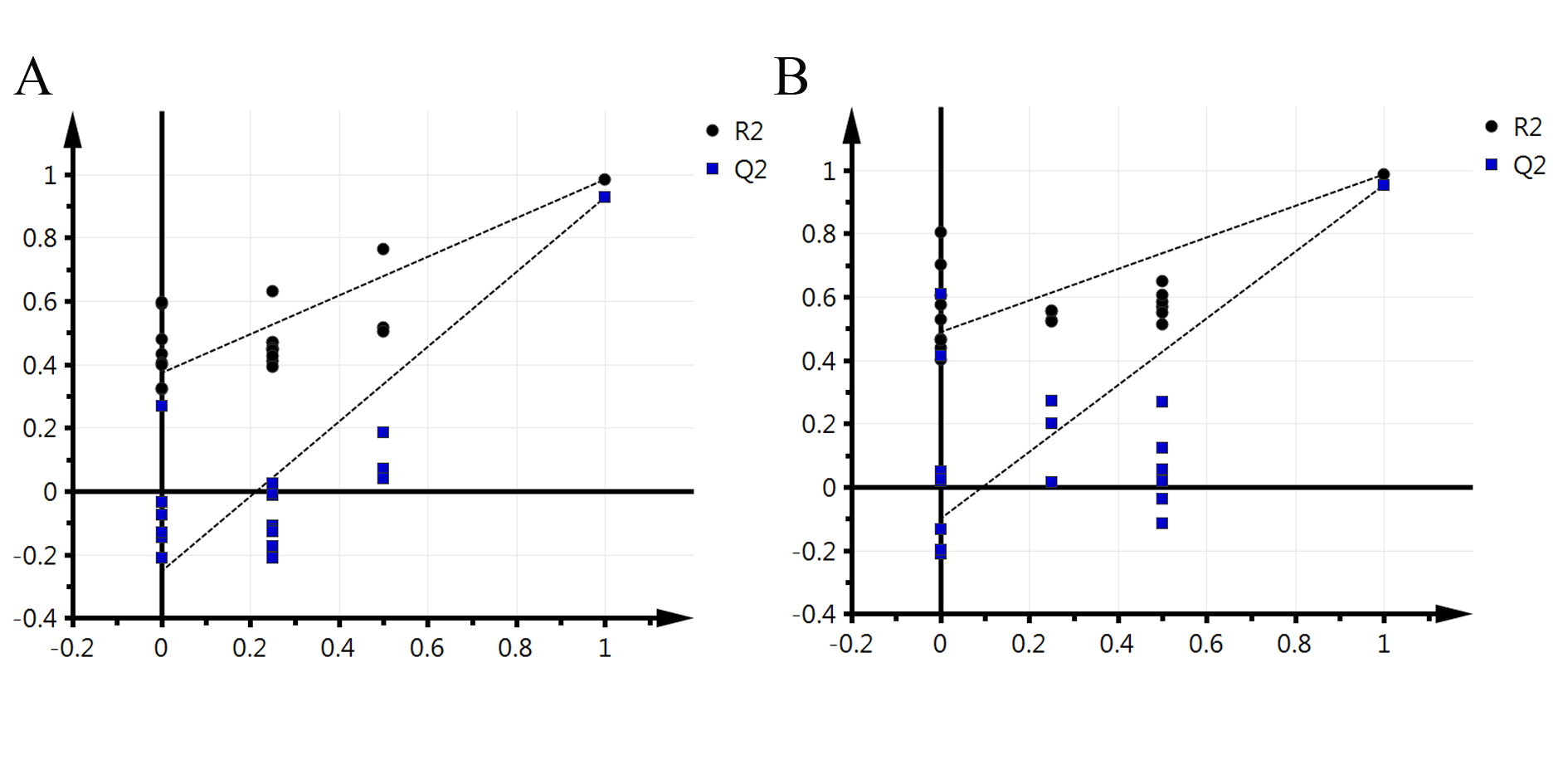

Supplement: S7 Fig — (A) C3H group and (B) G6PDx group. (TIF) [file pone.0151103.s007.tif]

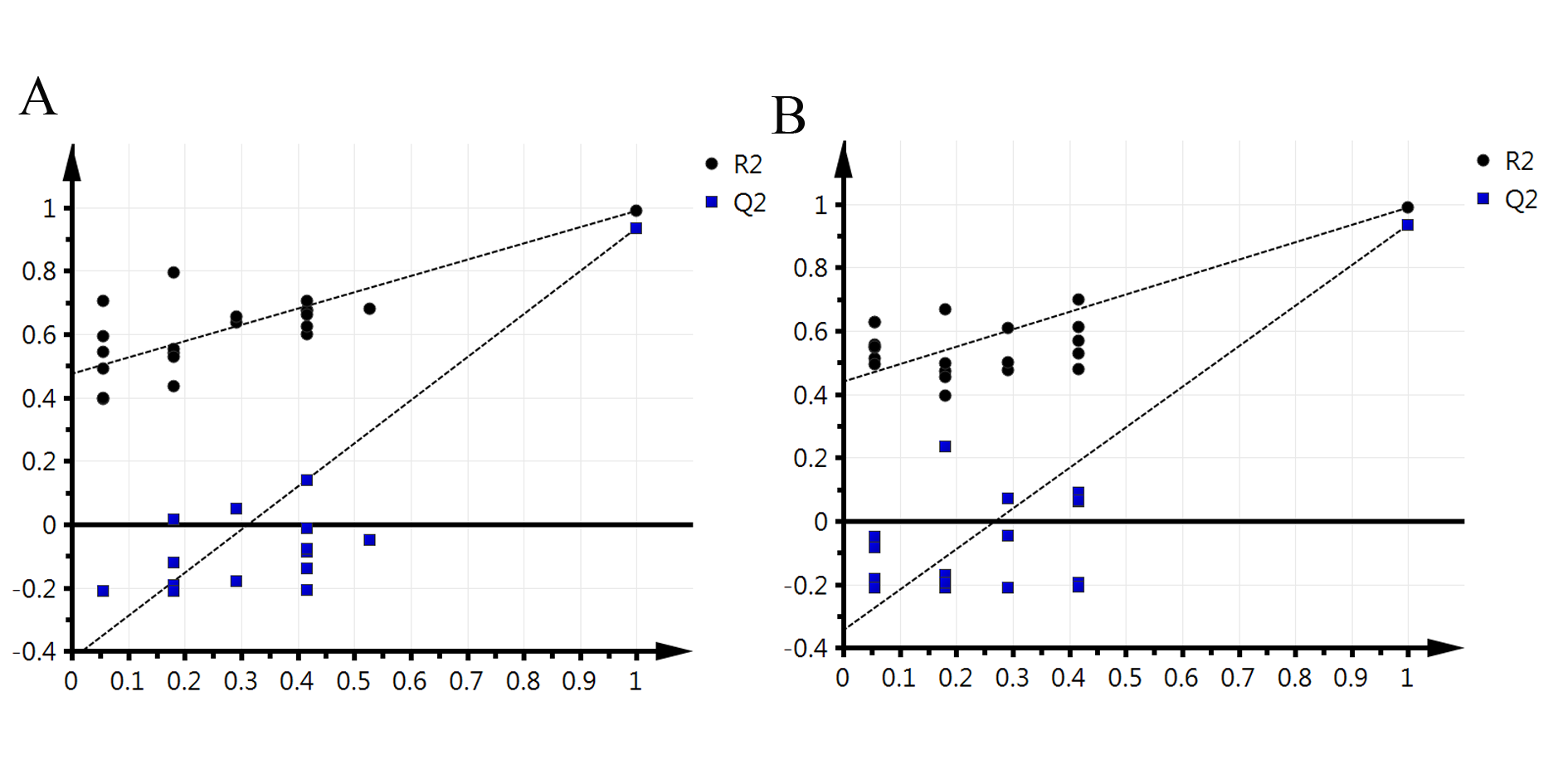

Supplement: S8 Fig — (A) C3H group and (B) G6PDx group. (TIF) [file pone.0151103.s008.tif]
